# TNFSF10/TRAIL regulates human T4 effector memory lymphocyte radiosensitivity and predicts radiation-induced acute and subacute dermatitis

**DOI:** 10.18632/oncotarget.7893

**Published:** 2016-03-16

**Authors:** Jan Baijer, Nathalie Déchamps, Hervé Perdry, Pablo Morales, Sarah Kerns, Alexandre Vasilescu, Sylvain Baulande, David Azria, Paul Henri Roméo, Annette Schmitz

**Affiliations:** ^1^ CEA/DRF/IRCM/SCSR, Fontenay-aux-Roses Cedex, France; ^2^ Inserm U967, Fontenay-aux-Roses Cedex, France; ^3^ Université Paris-Diderot, Paris, France; ^4^ Université Paris-Sud, Paris, France; ^5^ Université Paris-Saclay, Univ. Paris-Sud, Inserm, CESP, Villejuif, France; ^6^ University of Rochester Medical Center, Rochester, NY, USA; ^7^ ICGex Next-Generation Sequencing Platform, Institut Curie, Paris, France; ^8^ Department of Radiation Oncology, ICM-Val d'Aurelle and INSERM U896, Institut de Recherche en Cancérologie de Montpellier, Montpellier, France

**Keywords:** TRAIL, biomarker, lymphocyte, apoptosis, acute radiosensitivity

## Abstract

Sensitivity of T4 effector-memory (T4EM) lymphocytes to radiation-induced apoptosis shows heritability compatible with a Mendelian mode of transmission. Using gene expression studies and flow cytometry, we show a higher TNF-Related Apoptosis Inducing Ligand (*TRAIL/TNFSF10*) mRNA level and a higher level of membrane bound TRAIL (mTRAIL) on radiosensitive compared to radioresistant T4EM lymphocytes. Functionally, we show that mTRAIL mediates a pro-apoptotic autocrine signaling after irradiation of T4EM lymphocytes linking mTRAIL expression to T4EM radiosensitivity. Using single marker and multimarker Family-Based Association Testing, we identified 3 SNPs in the *TRAIL* gene that are significantly associated with T4EM lymphocytes radiosensitivity. Among these 3 SNPs, two are also associated with acute and subacute dermatitis after radiotherapy in breast cancer indicating that T4EM lymphocytes radiosensitivity may be used to predict response to radiotherapy. Altogether, these results show that mTRAIL level regulates the response of T4EM lymphocytes to ionizing radiation and suggest that *TRAIL/TNFSF10* genetic variants hold promise as markers of individual radiosensitivity.

## INTRODUCTION

Inter-individual differences in radiosensitivity are described since the discovery of the effects of ionizing radiation on human, and are now associated with toxicity that patients treated with radiotherapy may experience. Surprisingly, except for very rare monogenic diseases such as homozygous carriers of a mutated *ATM* gene [1], the genetic basis of individual radiosensitivity is poorly characterized. Numerous studies indicated a correlation between cellular- and clinical radiosensitivity as exemplified by the association between low CD8^+^-lymphocyte apoptosis and radiation induced late toxicity [2]. However, no consensus exists on available biological tests that can be reliably used for prediction of early- and/or late clinical adverse effects associated with radiotherapy [3, 4]. This absence of predictive tests for individual radiosensitivity together with deleterious side effects observed in a minority of patients treated by radiotherapy led to limitation of the radiation dose used in radiotherapy. Because of the direct relationship between radiation dose and tumor control, this limitation reduces efficacy of radiotherapy in the majority of patients. Thus, beyond technical developments such as optimizing radiation delivery, improving radiotherapy outcome requires a better understanding of the underlying mechanisms of individual radiosensitivity [5] that will ultimately allow personalized radiotherapy.

We studied human T-lymphocytes radiation-induced apoptosis to characterize any genetic contribution to radiosensitivity. Radiation induced apoptosis of CD4-positive T-lymphocytes lacking expression of both CD62L and CD45RA (T4EM lymphocytes) displayed significant heritability and transmission of this radiosensitivity phenotype within a cohort of large-kindred families was compatible with a simple Mendelian genetic model [6].

Genotype-phenotype correlations can be a consequence of genetically driven variation in gene expression that may be inherited [7]. Gene expression heritability within and across tissues revealed tissue- and/or cell-specific heritability [8] and trait-associated loci are enriched for expression quantitative trait loci (eQTL; [9]). In radio-genetics, understanding how genotype affects gene expression is an important goal that was in particular studied through the search for genetic markers among genes whose expression is modulated in response to irradiation [10, 11]. Most candidate gene approaches were centered on DNA damage, repair and signaling, but associations were not replicated, notably in the first genome-wide association studies to screen for SNPs in relation to radiotherapy toxicity and second malignant neoplasm [12, 13].

Here we show that the level of expression of *TRAIL/TNFSF10* is critically related to the previously defined cellular radiosensitivity phenotype. Functional studies showed that TRAIL blocking antibody, exogenous soluble TRAIL and soluble DR5 drastically reduce radiation-induced apoptosis and that membrane-bound TRAIL-mediated pro-apoptotic signaling is dependent on modulation of TRAIL shedding. Finally, we identified SNPs in the *TRAIL/TNFSF10* gene that are associated with the cellular radiosensitivity phenotype. Interestingly, two of those SNPs were genetically associated with a subset of acute clinical radiosensitivity toxicities in a cohort of breast cancer patients. The identification of the role of *TRAIL/TNFSF10* in susceptibility to radiation-induced apoptosis in human T4EM-lymphocytes suggests that the TRAIL-signaling pathway is associated with individual radiosensitivity and sheds new light on the role of *TRAIL/TNFSF10* in the response of the immune system to radiation.

## RESULTS

### *TRAIL/TNFSF10* mRNA level correlates with T4EM lymphocytes radiosensitivity

**Figure 1:**
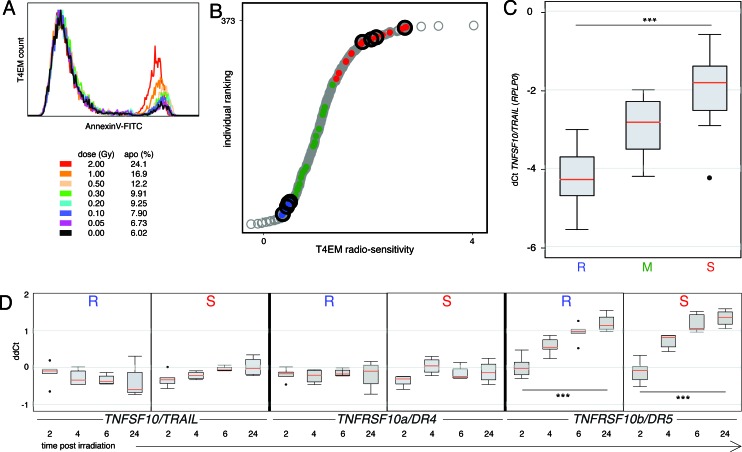
*TRAIL/TNFSF10* mRNA level correlates with T4EM lymphocytes radiosensitivity **A.** Dose dependent apoptosis of T4EM lymphocytes monitored by AnnexinV fluorescence intensity 18 hours after irradiation. **B.** Ranking of 373 individuals according to their T4EM lymphocytes radiosensitivity measured by the level of apoptosis (grey circles). T4EM lymphocytes from four “sensitive” and four “resistant” unrelated individuals (black circles) were selected for micro-array expression analysis. T4EM lymphocytes from fifteen “sensitive” (red), “median” (green) and “resistant” (blue) unrelated individuals were used for qPCR. **C.** Quantitative PCR analysis of expression level of *TRAIL/TNFSF10* in sorted T4EM lymphocytes from 15 “resistant” (R), 15 “median” (M), and 15 “sensitive” unrelated individuals (S). Results are shown as box plots of dCt with respect to the internal reference gene (*RPLP0*) with median values (red) (***p < 10^−3^). **D.** Multiplexed qPCR analysis of *TNFSF10/TRAIL* (left panels)*, TNFRSF10a/DR4* (middle panels) and *TNFRSF10b/DR5* (right panels) in sorted T4EM lymphocytes from 5 “resistant” (R) and 5 “sensitive” (S) unrelated individuals, 2, 4, 6, and 24 hours after irradiation at 2 Gy. Results are shown as box plots with median line (red) (***p < 10^−3^) that represent, for each time point post-irradiation (hours), the difference in dCt (ddCt) between non-irradiated sample and the dCt of the test sample.

The sensitivity of subpopulations of human T-lymphocytes to ionizing radiation-induced apoptosis was studied eighteen hours after irradiation (0-2 Gy) of PBMC samples of healthy blood donors using the previously defined radiosensitivity assay based on immunophenotyping and AnnexinV-labeling. Whereas the CD62L-positive T lymphocytes did not undergo apoptosis (Supplementary Figure 1), a dose-dependent increase of apoptosis was evidenced in the CD62L-negative, T4EM lymphocytes (Figure 1A). Exponential regression coefficients of dose-survival curves were used to classify human PBMC samples according to the radiosensitivity of their T4EM lymphocytes and radiosensitive and radioresistant samples were defined at the two ends of the T4EM lymphocytes radiosensitivity distribution.

To identify genes differentially expressed, array-based expression profiling of flow sorted T4EM lymphocytes of four radiosensitive and four radioresistant day-fresh samples was performed (Figure 1B; black circles). Using a radiosensitive to radioresistant ratio of more than 2 (or less than 0.5), an expression level at least 3 times over background and *p* < 0.01, we identified 31 genes expressed at a higher level (mean DF 2.40; 2.01 to 4.42), and 33 genes expressed at a lower level (mean DF −2.67; −2.01 to −7.48) in radiosensitive T4EM lymphocytes (Supplementary Table 1). Ontology analysis identified three pathways associated with radiosensitivity, cytokine-cytokine receptor interaction (hsa04060), immune response (GO:0006955), and death (GO:0016265) (Supplementary Table 2). Among the genes up-regulated in radiosensitive T4EM lymphocytes, *TRAIL/TNFSF10* displayed a 2.34-fold higher expression level, and was further studied. Quantitative PCR analysis of *TRAIL/TNFSF10* mRNA expression levels in flow sorted T4EM lymphocytes of “sensitive”, “median” and “resistant” samples (respectively illustrated by “red”, “green” and “blue” dots in the distribution of radiosensitivity phenotypes in Figure 1B) showed that *TRAIL/TNFSF10* mRNA level was 5.7-fold higher in radiosensitive compared to radioresistant T4EM lymphocytes, whereas *TRAIL/TNFSF10* mRNA levels of T4EM lymphocytes with “median” radiosensitivity were intermediate (Figure 1C). Only 1 out of 45 studied samples showed discordant T4EM lymphocytes radiosensitivity and *TRAIL/TNFSF10* expression level.

To study the effects of irradiation on TRAIL and TRAIL-receptors mRNA levels in T4EM lymphocytes, we performed multiplexed determination of their mRNA levels on independent triplicate sorts from radiosensitive and radioresistant T4EM lymphocytes at 2, 4, 6 and 24 hours after 2 Gy irradiation. Expression of the decoy receptor *TNFRSF10c/DcR1* was below detection limit (data not shown). No significant effect of irradiation on *TRAIL/TNFSF10*, on decoy receptor *TNFRSF10d*/*DcR2* (not shown), and on pro-apoptotic receptor *TNFRSF10a/DR4* mRNA levels was found in radiosensitive and radioresistant T4EM lymphocytes (Figure 1D, left and middle panels). In contrast, in both radioresistant and radiosensitive T4EM lymphocytes, mRNA levels of the pro-apoptotic receptor *TNFRSF10b/DR5* started to increase 4 hours after irradiation and remained high at 6 and 24 hours after irradiation (Figure 1D, right panels).

Altogether, these results indicate an association between TRAIL mRNA level and T4EM lymphocytes radiosensitivity and suggest a role for *TNRSF10b/DR5* in T4EM lymphocytes radiation induced apoptosis.

### Membrane bound TRAIL (mTRAIL) mediates pro-apoptotic autocrine signaling in irradiated T4EM lymphocytes

**Figure 2:**
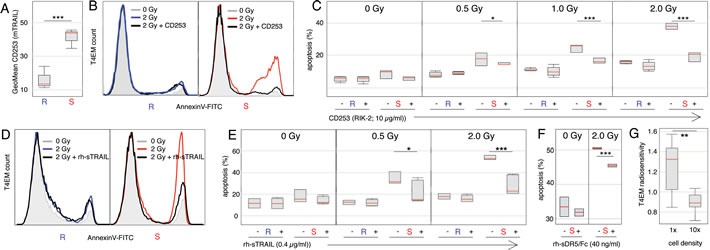
Membrane bound TRAIL (mTRAIL) mediates pro-apoptotic autocrine signaling in irradiated T4EM lymphocytes **A.** mTRAIL level, quantified by flow cytometry, on resting T4EM lymphocytes from 4 “sensitive” (S) and 4 “resistant” (R) samples from unrelated individuals. Results are shown as box plots with median line (red) (***p < 10^−3^). **B.** Apoptosis of T4EM lymphocytes monitored by AnnexinV fluorescence intensity of “resistant” (left panel) or “sensitive” (right panel) samples after 18 hours without irradiation (shaded grey), or after 2 Gy irradiation in the absence (“resistant” blue / “sensitive” red) or presence (black) of TRAIL blocking antibody (CD253). **C.** Percentage of apoptotic T4EM lymphocytes from 4 “sensitive” (S) and 4 “resistant” (R) samples, 18 hours after irradiation at 0, 0.5, 1, and 2 Gy, in the absence (−) or presence (+) of TRAIL blocking antibody (CD253). Results are shown as box plots with median line (red) (*p < 0.05, ***p < 10^−3^). **D.** Apoptosis of T4EM lymphocytes monitored by AnnexinV fluorescence intensity of “resistant” (left panel) or “sensitive” (right panel) samples after 18 hours without irradiation (shaded grey), or after 2 Gy irradiation in the absence (“resistant” blue / “sensitive” red) or presence of 0.4 μg/ml rh-sTRAIL (black). **E.** Percentage of apoptotic T4EM lymphocytes from 4 “sensitive” (S) and 4 “resistant” (R) samples, 18 hours after irradiation at 0, 0.5, and 2 Gy, in the absence (−) or presence (+) of rh-sTRAIL (0.4 μg/ml). Results are shown as box plots with median line (red) (*p < 0.05, ***p < 10^−3^). **F.** Percentage of apoptotic T4EM lymphocytes from 4 “sensitive” (S) samples, 18 hours after irradiation at 0 and 2 Gy, in the absence (−) or presence (+) of rh-sDR5/Fc (40 ng/ml). Results are shown as box plots with median line (red) (***p < 10^−3^). **G.** Radiosensitivity of 9 samples from unrelated “sensitive” individuals after 2 Gy irradiation at cell concentrations of 10^5^ /ml (1X) and 10^6^ /ml (10X) (**p < 10^−2^).

To show any involvement of TRAIL in T4EM lymphocytes radiosensitivity, we first analyzed TRAIL protein level in radiosensitive and radioresistant T4EM lymphocytes. TRAIL can be soluble (sTRAIL) or membrane-bound (mTRAIL) [14, 15]. Using sandwich ELISA detection, we could not detect any expression of soluble TRAIL (sTRAIL) indicating that the concentration of sTRAIL in the media was less than 20pg/ml of sTRAIL in the media. To determine the expression level of membrane bound TRAIL (mTRAIL), we used a monoclonal antibody panel comprising an anti-mTRAIL (CD253) antibody, and found a four-fold higher expression of mTRAIL on radiosensitive compared to radioresistant T4EM lymphocytes (Figure 2A and Supplementary Figure 2). Thus, radiosensitivity of T4EM lymphocytes is associated with expression level of mTRAIL.

To characterize the role of mTRAIL in radiation-induced apoptosis in T4EM, radiosensitive and radioresistant samples were irradiated in the presence of a soluble blocking antibody to human TRAIL (CD253; clone RIK-2). Apoptosis of T4EM lymphocytes in radioresistant samples was unaffected by the presence of the blocking antibody, whereas blocking of TRAIL resulted in strong inhibition of radiation-induced apoptosis of T4EM lymphocytes in radiosensitive samples, with levels of apoptosis in radiosensitive T4EM lymphocytes becoming similar to those observed in radioresistant T4EM lymphocytes, even after a 2.0 Gy irradiation (Figure 2B and 2C). Radiosensitive and radioresistant samples were then irradiated in the presence of recombinant human-soluble TRAIL (rh-sTRAIL), radiation induced apoptosis was inhibited in radiosensitive T4EM lymphocytes (Figure 2D and 2E). Inhibition of T4EM radiation-induced apoptosis was also observed when radiosensitive samples were irradiated in the presence of recombinant human soluble TRAIL DR5 Fc Chimera (rh-sDR5/Fc), a soluble form that antagonizes the pro-apoptotic receptor TNFRSF10b/DR5 [16]. This treatment decreased radiation-induced apoptosis of radiosensitive T4EM lymphocytes (Figure 2F). Finally, we studied any contribution of paracrine signaling in radiation-induced apoptosis of T4EM. At ten-fold higher cell concentrations, no increase in radiation-induced apoptosis could be detected, but on 9 “sensitive” samples, a ten-fold higher cell concentration resulted in 1.5-fold lower apoptosis-induction (Figure 2G).

Altogether, these results show that the radiation-induced apoptosis of T4EM lymphocytes is dependent on mTRAIL expression and suggest that mTRAIL mediates this radiation-induced apoptosis by an autocrine signaling involving the pro-apoptotic death receptors to TRAIL expressed by T4EM lymphocytes.

### 1,10-Phenanthroline increases mTRAIL expression level and induces T4EM apoptosis

**Figure 3:**
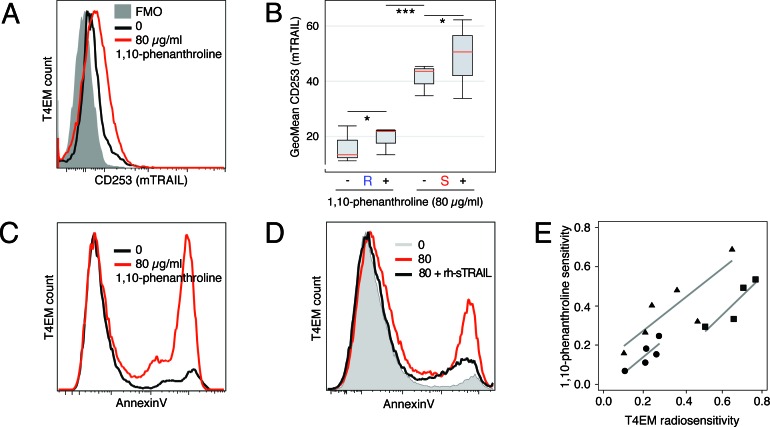
1,10-Phenanthroline increases mTRAIL expression level and induces T4EM lymphocytes apoptosis **A.** mTRAIL level, quantified by flow cytometry, on T4EM lymphocytes of a “sensitive” sample after 2 hours of treatment without (black) and with 80 μg/ml 1,10-phenanthroline (red). Corresponding FMO control histogram is shaded grey. **B.** mTRAIL expression on T4EM lymphocytes from 4 “sensitive” (S) and 4 “resistant” (R) samples two hours after treatment with 80 μg/ml of 1,10-phenanthroline. Results are shown as box plots with median line (red) (*p < 0.05 and ***p < 10^−3^). **C.** Apoptosis of T4EM lymphocytes, quantified by AnnexinV fluorescence, in a “sensitive” sample after two hours without (black) and with 80 μg/ml 1,10-phenanthroline (red). **D.** Apoptosis of T4EM lymphocytes, quantified by AnnexinV fluorescence, after 2 hours without (grey) or with 80 μg/ml 1,10-phenanthroline in the absence (red) or in the presence of 4 μg/ml rh-sTRAIL (black). **E.** Scatter plot of T4EM lymphocyte radiosensitivity (abscissa) versus T4EM lymphocyte sensitivity to 1,10-phenanthroline (ordinate). Three independent experiments were performed using 4 (cryopreserved; ■), 5 (fresh; •), and 6 (cryopreserved; ▲) samples. Regression lines were calculated for each experiment.

Whereas the proteases involved in the shedding of TRAIL are not well characterized, metalloprotease-inhibitor 1,10-phenanthroline was shown to reduce the shedding of TRAIL [17]. Two hours after 1,10-phenanthroline treatment, mTRAIL expression level was increased in both radioresistant and radiosensitive T4EM lymphocytes (Figure 3A). Nevertheless, mTRAIL expression always remained more than 2 fold lower in 1,10-phenanthroline treated radioresistant T4EM lymphocytes than in untreated radiosensitive T4EM lymphocytes (Figure 3B).

In the absence of irradiation, 1,10-phenanthroline induced apoptosis of B and T lymphocytes (data not shown). Specifically, T4EM lymphocytes underwent apoptosis 2 hours after treatment (Figure 3C). This apoptosis was mTRAIL dependent as it was inhibited when 1,10 phenanthroline treatment was performed in the presence of rh-sTRAIL (Figure 3D). Finally, using exponential regression coefficients of dose-survival curves on cryopreserved samples or fresh PBMC samples exposed to 1,10 phenanthroline or to ionizing radiation, we showed that radiosensitivity and sensitivity to 1,10-phenanthroline were positively correlated (Figure 3D).

### Association of *TRAIL/TNFSF10* SNPs with radiosensitivity and radio-induced acute and subacute dermatitis

**Figure 4:**
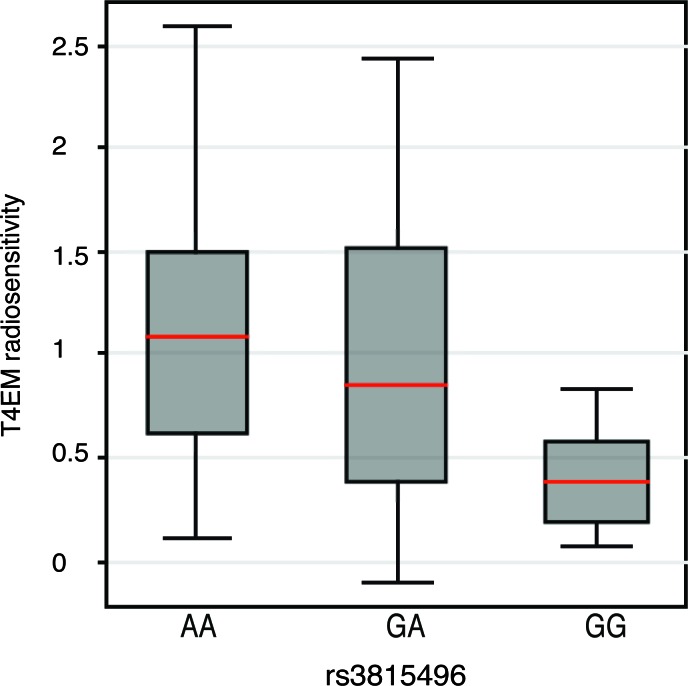
T4EM lymphocyte radiosensitivity monitored by rs3815496 genotype Association between T4EM lymphocyte radiosensitivity and genotype at the rs3815496 SNP of the *TRAIL/TNFSF10-*gene. Results are shown as box plots with median line (red) for 126 unrelated individuals by genotype at rs3815496; AA (*n* = 65), GA (*n* = 55), and GG (*n* = 6).

To investigate any genetic association between the *TRAIL/TNFSF10* locus and radiosensitivity of T4EM lymphocytes, coding- and flanking regions of the *TRAIL/TNFSF10* gene that contain SNPs that might be associated to- or act as eQTLs, were genotyped. A 4.2 kb region encompassing the *TRAIL/TNFSF10* gene from 373 individuals previously characterized for T4EM lymphocytes radiosensitivity and subjected to heritability and segregation analysis [6] was sequenced. Supplementary Table 3 summarized results for the 36 polymorphic markers identified, with their frequencies and positions in the studied population. Single marker association analysis between the 15 identified frequent variants (MAF > 5%) and T4EM radiosensitivity by Family-Based Association Testing (FBAT) showed a significant association for 3 SNPs; rs3815496 (*p* = 0.03; intron 3, MAF = 0.28), rs1131532 (*p* = 0.04; exon 5, MAF = 0.28), and rs1131535 (*p* = 0.05; 3′ UTR exon 5, MAF = 0.38). SNPs rs1131532 and rs3815496 were in strong linkage disequilibrium, and in partial linkage with rs1131535. Several other SNPs in the sequenced region were found to be marginally but significantly associated (Table 1). Multi-Marker association testing between the 15 most frequent SNPs of the *TNFSF10/TRAIL* gene and T4EM lymphocytes radiosensitivity was shown significant by the Family based MM association test (FBAT-MM) (*p* = 0.02) and by Fisher product (*p* = 0.04), demonstrating a genetic contribution to the T4EM lymphocytes radiosensitivity in or close to the *TRAIL/TNFSF10* gene. The analysis of a selection of 126 unrelated individuals, using T4EM lymphocytes radiosensitivity median values by genotype at rs3815496 identified A as the risk allele for high levels of radiation-induced apoptosis in T4EM (Figure 4).

**Table 1:** Single point association analysis (FBAT) between 15 frequent SNP of *TRAIL/TNFSF10* and radiosensitivity of *T4EM*

Location	SNP	*p*
Chr3:172,241,890	rs12488654	0.08
Chr3:172,241,866	rs365238	0.35
Chr3:172,241,759	rs3136586	0.08
Chr3:172,240,999	rs2270418	0.08
Chr3:172,229,871	rs2241063	0.11
Chr3:172,228,544	rs3136597	0.24
Chr3:172,227,199	rs3815496	0.03
Chr3:172,224,771	rs17848019	0.06
Chr3:172,224,303	rs1131532	0.04
Chr3:172,224,075	rs1131535	0.05
Chr3:172,223,926	rs1131542	0.06
Chr3:172,223,690	rs1131568	0.09
Chr3:172,223,627	rs1131579	0.11
Chr3:172,223,620	rs1131580	0.09
Chr3:172,223,376	rs11720451	0.09

Association between the identified *TRAIL/TNFSF10* SNPs and CD8^+^ lymphocyte apoptosis as well as with clinical radiosensitivity endpoints was studied in a set of 113 genotyped breast cancer patients, included in the Co-Ho-RT study [18] (Table 2). Blood samples from these patients are characterized for radiation-induced CD8^+^ T-lymphocyte apoptosis, according to Ozsahin [2]. Genetic association study indicated a trend towards association of the three SNPs, rs3815496 (MAF = 0.28, *p* = 0.06), rs1131532 (MAF = 0.28, *p* = 0.06), and rs1131535 (MAF = 0.44, *p* = 0.09), with radiation-induced CD8^+^ T-lymphocyte apoptosis. Finally, acute and subacute dermatitis (10 cases), acute erythema (44 cases), acute hyperpigmentation (7 cases), late fibrosis (28 cases), and late telangiectasia (3 cases) were evaluated for association with these three *TRAIL/TNFSF10* SNPs. None of these three SNPs was associated with late fibrosis, acute erythema, or acute hyperpigmentation. An association was shown with late telangiectasia but, due to insufficient number of cases, this result was not considered. Interestingly, for acute and subacute dermatitis, a strong association was identified with rs3815496 (*p* = 0.047, effect allele = A, OR: 3.88, 95% c.i: 0.87-17.22), and with rs1131532 (*p* = 0.045, effect allele = C, OR 3.90, 95% c.i: 0.88-17.30), but not with rs1131535 (Table 2).

**Table 2:** Association testing between *TRAIL/TNFSF10* SNP and CD8+ lymphocyte apoptosis and radiotherapyinduced skin reaction

Endpoint	rs1131535 (effect allele = A, MAF = 0.44)	rs1131532 (effect allele = C, MAF = 0.28)	rs3815496 (effect allele = A, MAF = 0.28)
**CD8+ lymphocyte apoptosis**	1.84 (−0.24, 3.93) *p* = 0.086	2.09 (−0.09, 4.27) *p* = 0.063	2.08 (−0.10, 4.26) *p* = 0.064
**Acute & subacute dermatitis** (10 cases, 103 controls)	1.58 (0.63, 3.96) *p* = 0.336	3.90 (0.88, 17.30) *p* = 0.045	3.88 (0.87, 17.22) *p* = 0.047
**Acute erythema** (44 cases, 69 controls)	0.78 (0.45, 1.34) *p* = 0.364	0.69 (0.39, 1.25) *p* = 0.252	0.69 (0.38, 1.24) *p* = 0.244
**Acute hyperpigmentation** (7 cases, 106 controls)	1.71 (0.57, 5.09) *p* = 0.338	1.49 (0.40, 5.52) *p* = 0.560	1.48 (0.40, 5.50) *p* = 0.566
**Late fibrosis** (28 cases, 83 controls)	1.10 (0.84, 1.43) *p* = 0.486	1.00 (0.76, 1.31) *p* = 0.987	1.00 (0.76, 1.31) *p* = 0.975
**Late telangiectasia** (3 cases, 108 controls)	6.61 (0.76, 57.56) *p* = 0.045	2.02 (0.23, 17.67) *p* = 0.517	2.01 (0.23, 17.59) *p* = 0.521

Association (SNPTEST) was evaluated in a set of 113 genotyped breast cancer patients, included in the Co-Ho-RT study.

Altogether, these results show a genetic association between the *TRAIL/TNFSF10* locus and radiosensitivity of T4EM lymphocytes and suggest that *TRAIL/TNFSF10* genetic variants might be used as markers of individual radiosensitivity.

## DISCUSSION

Advances in radiotherapy treatment suffer from the lack of validated tests and genetic markers that can be used to predict individual radiosensitivity. Here we show that the expression level of *TRAIL/TNFSF10* in resting T4EM lymphocytes identifies individuals with a high, TRAIL dependent, apoptotic response to ionizing radiation of T4EM lymphocytes.

The *TNFRSF10b/DR5* receptor regulates radiation-induced apoptosis in mice and in breast cancer cell lines [19-21]. We now show its increased expression in T4EM lymphocytes after irradiation, suggesting that this receptor might play a broader role in radiation-induced apoptosis. Whereas in TRAIL sensitive cells, adding recombinant human soluble TRAIL (rh-sTRAIL) results in apoptosis [22], we show an inhibiting effect of rh-sTRAIL and of TRAIL blocking antibody on the radiation-induced apoptosis of T4EM lymphocytes. Together with the apoptosis-reducing effect observed at higher cell concentrations and the increased expression of membrane-bound (mTRAIL) in radiosensitive T4EM lymphocytes, these results indicate that mTRAIL but not sTRAIL expression on T4EM regulates their radiation-induced apoptosis. Finally, as DR5 but not mTRAIL expression increased after irradiation, these results suggest a paracrine, mTRAIL dependent, activation of mTRAIL/DR5 signaling after irradiation of T4EM lymphocytes, and pinpoint mTRAIL and *TRAIL/TNFSF10* expression level as markers of T4EM radiosensitivity.

After 1,10-phenanthroline treatment, the increased level of mTRAIL in T4EM lymphocytes showed the effects of metalloproteases on TRAIL shedding [17] in these cells. Interestingly, mTRAIL expression level in radioresistant T4EM lymphocytes treated with 1,10-phenanthroline was still more than two times lower than in untreated radiosensitive T4EM lymphocytes. Therefore, differences in mTRAIL shedding between resistant and sensitive individuals do not account for differential mTRAIL protein level, suggesting that *TRAIL/TNFSF10* mRNA level may regulate mTRAIL expression in T4EM lymphocytes.

Measurements of radiosensitivity using only cell based assays have faced uncontrolled experimental variability [23]. Here we show an association between T4EM lymphocytes radiosensitivity and three SNPs within the *TRAIL/TNFSF10-*gene. Interestingly, these same SNPs were associated with risk and/or treatment outcome in ovarian and breast cancer [24-26]. Further investigation is now needed to understand if and/or how these genetic variants influence *TRAIL/TNFSF10* mRNA level.

CD8^+^ lymphocyte apoptosis was shown to predict radiation-induced late toxicity [2] and this correlation was recently extended using a prospective multicenter study [27]. Although we did not detect a strong association between the three *TRAIL/TNFSF10* SNPs characterized in this study and CD8^+^ lymphocyte apoptosis, a trend towards such an association was evidenced indicating the need for further association studies using other cohorts.

No association between the *TRAIL/TNFSF10* SNPs and late fibrosis could be detected. However, the importance of TRAIL in the clinical response to radiation and in its physiopathology was suggested by the association between two of the three reported *TRAIL/TNFSF10* SNPs and acute and subacute radio-induced dermatitis. Acute and subacute dermatitis are a subset of radiotherapy-induced skin reactions (RISR) that are characterized by acute skin injury associated with inflammation, and are graded in the CTCAE system. These dermatitides can also occur after coronary angiography or embolization procedures [28]. Thus, the SNPs characterized in this study might be useful both in radiotherapy and interventional procedures.

Altogether our study highlights a role of TRAIL in T4EM-lymphocyte radiation induced apoptosis and the association of *TRAIL/TNFSF10* SNPs with subtypes of radio-induced dermatitis. International consortium research that will provide the power of GWAS will now enable to appreciate the relative contribution of the identified *TRAIL/TNFSF10* SNPs to radiotherapy toxicity [29, 30].

## MATERIALS AND METHODS

### Healthy donors and samples

All samples were prepared from venous blood drawn from healthy normal volunteers between 9:00 am and 11:00 am. Their inclusion was preceded by a medical interview and informed written consent was obtained from all donors, in accordance with local ethics guidelines at Fondation Jean Dausset-CEPH or Etablissement Français du Sang, Hôpital Saint Louis, Paris.

### Samples

Peripheral blood mononuclear cells (PBMC) were prepared from fresh (within 6h, average 180 min) anticoagulated blood samples, by standardized procedures, as previously described [31]. Briefly, 30 ml of two-fold diluted blood were underlayered with 10 ml of Histopaque^®^-1077 (Sigma-Aldrich), and centrifuged at 2,000*g* for 20 minutes. The collected interlayer was washed twice with 40 ml of RPMI1640/15% Fetal Calf Serum (FCS), and used as further described, or collected in FCS/10% DMSO for cryopreservation. Fractions of 1 ml each, corresponding to 4 ml of blood, were immediately frozen, using controlled temperature decrease, and stored in liquid nitrogen. Thawing of cells was performed by rapid transfer of cryotubes to 56°C waterbath (45 sec), addition of 1 ml of FCS and transfer of contents to 12 ml of RPMI/15% FCS. Thawed cells were washed twice, to be finally resuspended in the appropriate volume of culture medium (RPMI1640/15% FCS).

DNA extractions were performed from PBMC by classical phenol/chlorophorm procedures. Yield and purity were assessed spectrophotometrically at 260 and 280 nm (Thermo Scientific NanoDrop™ 1000). Familial relations were recorded from oral interview and HLA-A sequencing was used to exclude from family-based association analysis those samples presenting incompatibilities with announced familial interrelations.

### Monoclonal antibodies and proteins

All monoclonal antibodies were used at saturating conditions, as determined by titrations. Anti-human CD antibodies came from Becton Dickinson ImmunoCytometry Systems (CD62L-PE, CD62L-PECy5.1, CD14-PECy5.1, CD-235a-PECy5.1, CD27-PECy5.1, CD4-PECy7, CD19-PECy7, CD45RA-APC, CD8-APCH7), from Beckman Coulter (CD3-PETxR) from Biolegend (CD253/TRAIL-PE, CD62L-BV421, CD3-BV605), or from R&D Systems (CD262/DR5-APC). Recombinant human soluble TRAIL (rh-sTRAIL; Cat# 375-TL), anti-TRAIL cytotoxicity blocking antibody (CD253; clone RIK-2) and recombinant human soluble DR5 Fc chimera (rh-sDR5/Fc; Cat#631-T2) were from R&D Systems, AnnexinV-FITC from Becton Dickinson, and 1,10-phenanthroline from Sigma-Aldrich.

### Apoptosis induction

Cell suspensions at approximately 5×10^5^ cells/ml (or at cell densities specified in the results section) in RPMI1640/15% FCS, were irradiated at a dose rate of approximately 0.5 Gy/min with a ^137^Cs-source (IBL637; Cis-Bio international/Scherring, Saclay, France), and were incubated in the presence of indicated concentrations of rh-sTRAIL, CD253, rh-sDR5/Fc, or of 1,10-phenanthroline (stock solution of 200 mM in DMSO). All experiments described were performed with the same batch of heat inactivated FCS, previously tested for compatibility with our experimental procedures. Incubations of 18 hours or less as specified in results section were at 37°C and 5% CO2.

### Cell labeling for lymphocyte subset identification and apoptosis quantification

Treated cells were collected and washed with AnnexinV buffer (140mM NaCl, 5mM CaCl2, 10mM HEPES; ph7.4), and stained with 2.5 μl AnnexinV-FITC in combination with fluorochrome-conjugated CD markers in 100ul for 30 min. For cell sorting for gene expression analysis, Annexin V positive cells were excluded, and CD3 labeling was omitted to avoid TCR-mediated activation signaling. Furthermore, CD14 and CD235a were included to exclude monocytes and reticulocytes susceptible to contaminate the sort gates. CD27 was included for sorts of cryopreserved samples to avoid contamination of sort gates by central memory lymphocytes that transiently lost CD62L through sample manipulation procedures. For CD253 (mTRAIL) quantification, a compact panel designed to limit the need for compensation including AnnexinV, CD253, CD3, CD4, and CD62L was used.

### Flow cytometry

Cells were washed in AnnexinV-buffer and just prior flow cytometry, 50 μL of AnnexinV-buffer containing 0.2 μg/mL Hoechst 33258 (Molecular Probes, Eugene, OR), was added for dead cell exclusion. Samples were analyzed using a three-laser CyAn LX system, (DakoCytomation, Fort Collins, CO) or a 5-laser SORP LSRII (Becton Dickinson). Cell sorting was performed using a custom-built five-laser InFlux system (Becton Dickinson). All filters were from Chroma Technologies Inc., Brattleboro, Vermont, and all fluorescence signals were log-amplified, and stored in listmode. CompBeads (Becton Dickinson) were used to assist in determination of spill-over in every individual experiment (CD3-FITC was used instead of AnnexinV-FITC) and Fluorescence Minus One (FMO) controls were used where needed, in particular for mTRAIL quantifications (SortWare; Becton Dickinson or Flowjo version 9.6.4 ; Treestar, Ashland, OR). All sorts were performed in single droplet count mode, with an extended coincidence mask of one-and-a-half droplet. Doublet exclusion was performed by gating on the triggering parameter (FSC) *versus* time of flight.

### Data analysis

Data was analyzed using FlowJo. Listmode file analysis proceeded through the exclusion of non-lymphocyte events, predominantly monocytes, by using a large scattergate, shaped to include apoptotic lymphocytes (lower FSC, higher SSC). Subsequently, dead cells were excluded on the basis of HO33258 fluorescence. T Lymphocytes were identified on the basis of positive PETxR (CD3) fluorescence. A PECy7 (CD4) *versus* APCH7 (CD8) histogram allowed identification of CD4-positive T lymphocytes (T4) and CD8-positive T lymphocytes (T8). An APC (CD45RA) *versus* PE (CD62L) histogram discriminated between naive (CD62L+CD45RA+), central memory (CM; CD62L+CD45RA-), effector memory (EM; CD62L-CD45RA-), and terminal effector (TE; CD62L- CD45RA+) T4 and T8. The proportion of apoptotic cells was determined by application of an identical gate on a bivariate plot of FSC *versus* FITC fluorescence (AnnexinV) to all of the identified subpopulations. Dose-effect curves were generated using at least four doses, and used for the quantitative evaluation of 1,10-phenanthroline sensitivity and radiation sensitivity, as previously described [31].

### Microarray cDNA hybridization analysis

Cells of interest, recovered by centrifugation directly after sort, were lysed by RLT buffer (Qiagen) and cryopreserved until RNA extraction. Total RNA was extracted from a minimum of 300,000 cells using the RNeasy Plus Micro Kit (Qiagen). RNA samples were re-suspended in RNase-free water and stored at −80°C. RNA yield and purity were assessed spectrophotometrically at 260 and 280 nm (Thermo Scientific NanoDrop™ 1000). RNA integrity was evaluated using a Bioanalyzer 2100 (Agilent). Microarray experiments and part of data analysis were performed by PartnerChip (Evry, France) following the Affymetrix-recommended procedures. Target was prepared from 4 sensitive and 4 resistant individuals. Targets were pooled after individual quality control in equal proportions and hybridized on a HU133 2.0 plus array (54,645 probe sets) according to the Affymetrix Two-Cycle technical protocol. Fluorescent images were detected in a GeneChip Scanner 3000 (Affymetrix). Expression data and raw expression data (CEL files) were generated using GCOS software (Affymetrix). Quality control was assessed based on 3′/5′ ratios of glyceraldehyde 3-phosphate dehydrogenase (GAPDH) and β-actin control probe sets.

### Real-time quantitative PCR gene expression analysis

Triplicate sorts for qPCR gene expression studies were performed by direct deposition of cells in MicrAmp optical tubes (Applied Biosystems), containing 15 μl of one step RT-qPCR mix. RT-qPCR pre-amplification mix contained Platinum Taq polymerase and SuperScript III reverse transcriptase (Invitrogen), a mixture of Taqman primer-probes, specific for the transcripts of interest (Supplementary Table 4; Applied Biosystems) at 0.2X concentration, CellsDirect One-Shot qRT-PCR buffer (Invitrogen), and SuperaseIn RNase inhibitor. Immediately following cell sorting, samples were thoroughly mixed, and reverse transcribed (55°C for 10 min, 50°C for 50 min), and pre-amplified (14 cycles of PCR at 95°C for 15 sec and 62°C for 45 sec). After pre-amplification, samples were diluted ten-fold, for gene-specific quantitative PCR (35 cycles of 95°C/15 sec, 62°C/45 sec) on a HT7900 (Applied Biosystems). All primers were standard Taqman assays, and reaction conditions were according to manufacturers indications. Results were expressed as the difference between the Ct of the internally amplified reference gene (VIC-labeled *RPLP0*, *ACTB*, or *GAPDH*), and the FAM-labeled test gene, averaged over triplicate sorts, and termed dCT. Assay performance control on cryopreserved lymphocytes included mean dCt on different cryopreserved vials from same blood sample, mean dCt from independent blood samples from same individual. Samples from reference individuals were included in each experiment. Radiation induction of expression is expressed as the difference between dCT at dose 2.0 Gy and dCt at dose 0 Gy, for each timepoint after irradiation, and was termed ddCT.

### DNA genotyping

Primers were established in order to amplify by PCR the exon-containing DNA fragments and the promoters (Supplementary Tables 5 and 6). The PCRs were performed in a 15 μL reaction mixture containing 25 ng of DNA. Sequencing reactions were performed according to the dye terminator method using an ABI PRISM^®^ 3730xl DNA Analyzer (Applied Biosystems, Foster City, CA, USA). Alignment of experimental results, SNP discovery and genotyping were performed with the software Genalys. The genomic sequence used for the alignment is *TRAIL/TNFSF10* (NC_000003.11). The genotypes for rs3136597 and rs2241063 were obtained by Taqman technology, assays C__27464917_20 and C___3260973_20 respectively (Applied Biosystems).

### Family based association testing of frequent variants (FBAT)

The FBAT method and software [32, 33] was used to perform a single marker analysis between the frequent SNPs and the quantitative trait while taking into account the family structure (command fbat -o). Two methods have been used to obtain a *p-*value for the global association of *TNFSF10* with the trait : a) The Fisher product [34] of the *p-*values obtained from the single point association analysis was taken, and its significance was assessed using 10,000 permutations, b) The FBAT Multi-Marker statistic [35, 36] was computed, and its significance was assessed using 10,000 permutations.

### Radiotherapy induced skin reaction association testing with *TRAIL/TNFSF10* SNPs (SNPTEST)

Acute and subacute dermatitis, acute erythema, acute hyperpigmentation and late fibrosis were assessed prospectively using the CTCAE v3 grading system in the CO-HO-RT trial [18]. Toxicity was treated as a binary outcome and was determined by taking the highest CTCAE grade occurrence during radiotherapy to 6 weeks after radiotherapy for acute toxicities and from 3 months after radiotherapy to the end of follow-up (minimum 2 years, maximum 8 years) for late toxicities. For each toxicity endpoint, patients were considered cases if they had CTCAE grade ≥ 2 and controls if they had CTCAE grade ≤ 1. The percentage of CD8+ lymphocytes undergoing radiation-induced apoptosis was treated as a continuous outcome.

Genomic DNA was isolated from blood, genotyped using a commercial genome-wide SNP array (Affymetrix SNP6.0, Affymetrix, San Diego, CA)]. Because rs1131532, and rs3815496 were not directly genotyped on the array, the surrounding region of chromosome 3 (position 100,000,001 to 200,000,000, hg build 19) was imputed to the 1000 Genomes reference data using IMPUTE2 software (37]. Following imputation, the SNPs of interest were confirmed to be in Hardy-Weinberg equilibrium (*p*-value > 10^−6^) and showed good imputation quality (info score > 0.3). SNPTEST software [38] was used to analyze SNP-phenotype association with the frequentist test and expected method, which uses expected genotype counts (i.e. genotype dosages) obtained from imputation. An additive genetic inheritance model was assumed in all analyses.

### Statistical analysis

Statistical analyses and data presentation were performed with Stata (Stata Corp., College Station, TX). In box plot representations of results, boxes extend from 25^th^ to 75^th^ percentiles, with whiskers indicating upper- and lower adjacent values (1.5 times interquartile range). Each box represents at least three independent experiments.

## SUPPLEMENTARY INFORMATION TABLES AND FIGURES


