# Supplementary material for: TNFSF10/TRAIL regulates human T4 effector memory lymphocyte radiosensitivity and predicts radiation-induced acute and subacute dermatitis
Source: Oncotarget. 2016 Mar 16;7(16):21416–27. doi: 10.18632/oncotarget.7893 (PMC5008295; doi:10.18632/oncotarget.7893)
Supplement: Supplementary file 1 [file oncotarget-07-21416-s001.pdf]

TNFSF10/TRAIL regulates human T4 effector memory lymphocyte radiosensitivity and predicts radiation-induced acute and subacute dermatitis

Supplementary Information

Supplementary Table 1 : Differentially expressed genes in T4EM lymphocytes from sensitive versus resistant samples

Gene Symbol	Description	Sensitive / Resistant (Fold-change)
CCR10	chemokine (C-C motif) receptor 10	**4,42**
DHPS	deoxyhypusine synthase	**3,34**
MALAT1	metastasis associated lung adenocarcinoma transcript 1 (non-protein coding)	**3,31**
NEAT1	non-protein coding RNA 84	**3,17**
GZMH	granzyme H (cathepsin G-like 2, protein h-CCPX)	**2,86**
USP34	ubiquitin specific peptidase 34	**2,85**
AP2S1	adaptor-related protein complex 2, sigma 1 subunit	**2,81**
IGLV2-14	immunoglobulin lambda variable 2-14	**2,61**
RPL7AP68	ribosomal protein L7a pseudogene 68	**2,59**
RAB1B	RAB1B, member RAS oncogene family	**2,48**
DNAJC21	DnaJ (Hsp40) homolog, subfamily C, member 21	**2,47**
IL2RG	interleukin 2 receptor, gamma (severe combined immunodeficiency)	**2,41**
GBP3	guanylate binding protein 3	**2,36**
TNFSF10	tumor necrosis factor (ligand) superfamily, member 10	**2,34**
STAT1	signal transducer and activator of transcription 1, 91kDa	**2,28**
SLC4A7	solute carrier family 4, sodium bicarbonate cotransporter, member 7	**2,27**
FKSG49	FKSG49	**2,24**
BCLAF1	similar to Bcl-2-associated transcription factor 1 (Btf); BCL2-associated transcription factor 1	**2,24**
LAIR2	leukocyte-associated immunoglobulin-like receptor 2	**2,21**
RNPS1	similar to ribonucleic acid binding protein S1; RNA binding protein S1, serine-rich domain	**2,21**
GBP1	guanylate binding protein 1, interferon-inducible, 67kDa	**2,18**
RAC2	ras-related C3 botulinum toxin substrate 2 (rho family, small GTP binding protein Rac2)	**2,17**
AAGAB	alpha- and gamma-adaptin-binding protein p34	**2,16**
MTM1	myotubularin 1	**2,15**
POGZ	pogo transposable element with ZNF domain	**2,14**
TXNDC17	thioredoxin domain containing 17	**2,12**
AAK1	AP2 associated kinase 1	**2,11**
SLC9A3R1	solute carrier family 9 (sodium/hydrogen exchanger), member 3 regulator 1	**2,09**
GZMM	granzyme M (lymphocyte met-ase 1)	**2,05**
STOM	stomatin	**2,03**
ASCC1	activating signal cointegrator 1 complex subunit 1	**2,02**
SYTL2	synaptotagmin-like 2	**2,02**
ZRANB2	zinc finger, RAN-binding domain containing 2	**2,01**

SLC25A33	solute carrier family 25, member 33	**-2,01**
AEN	apoptosis enhancing nuclease	**-2,02**
CLP1	CLP1, cleavage and polyadenylation factor I subunit, homolog (S. cerevisiae)	**-2,02**
ZNF236	zinc finger protein 236	**-2,02**
LOC654433	hypothetical LOC654433	**-2,05**
SVIP	small VCP/p97-interacting protein	**-2,06**
EMR1	egf-like module containing, mucin-like, hormone receptor-like 1	**-2,09**
MS4A6A	membrane-spanning 4-domains, subfamily A, member 6A	**-2,09**
KCTD12	potassium channel tetramerisation domain containing 12	**-2,09**
FCGRT	Fc fragment of IgG, receptor, transporter, alpha	**-2,1**
SIK1	salt-inducible kinase 1	**-2,11**
FAM69A	family with sequence similarity 69, member A	**-2,14**
ZNF331	zinc finger protein 331	**-2,24**
NEFL	neurofilament, light polypeptide	**-2,24**
SLC35C2	solute carrier family 35, member C2	**-2,25**
HBB	hemoglobin, beta	**-2,25**
PCF11	PCF11, cleavage and polyadenylation factor subunit, homolog (S. cerevisiae)	**-2,26**
HBA1 / HBA2	hemoglobin, alpha 2; hemoglobin, alpha 1	**-2,34**
G0S2	G0/G1switch 2	**-2,35**
TM2D2	TM2 domain containing 2	**-2,46**
IER3	immediate early response 3	**-2,67**
NR4A2	nuclear receptor subfamily 4, group A, member 2	**-2,83**
HLA-DQB1	major histocompatibility complex, class II, DQ beta 1; similar to major histocompatibility complex, class II, DQ beta 1	**-2,95**
ASAH1	N-acylsphingosine amidohydrolase (acid ceramidase) 1	**-3,4**
SULT1A1	sulfotransferase family, cytosolic, 1A, phenol-preferring, member 1	**-3,48**
SPATA24	hypothetical protein LOC202051	**-3,52**
SGK1	serum/glucocorticoid regulated kinase 1	**-3,58**
RGS1	regulator of G-protein signaling 1	**-3,69**
MYOM2	myomesin (M-protein) 2, 165kDa	**-4,17**
IL8	interleukin 8	**-4,37**
IL1B	interleukin 1, beta	**-7,48**

SupplementaryTable 2 : Ontology analysis

Category	Term	PValue	Count	%	Fold Enrichment	Genes
hsa04060	Cytokine-cytokine receptor interaction	2,45E-03	5	7,46	7,81	TNFSF10, IL8, CCR10, IL1B, IL2RG
GO:0006955	immune response	4,00E-03	9	3,39	14,06	HLA-DQB1, TNFSF10, RGS1, IL8, IL1B, IL2RG, FCGRT, GBP3, GBP1
GO:0016265	death	5,34E-03	9	3,23	14,06	GZMM, IER3, SGK1, TNFSF10, AEN, NR4A2, IL1B, GZMH, STAT1
hsa04062	Chemokine signaling pathway	8,42E-03	4	8,37	6,25	RAC2, IL8, CCR10, STAT1
GO:0008219	cell death	1,76E-02	8	2,89	12,5	GZMM, IER3, SGK1, TNFSF10, AEN, IL1B, GZMH, STAT1
GO:0007626	locomotory behavior	1,95E-02	5	4,75	7,81	RAC2, IL8, CCR10, NR4A2, IL1B
GO:0042330	taxis	2,23E-02	4	6,5	6,25	RAC2, IL8, CCR10, IL1B
GO:0006935	chemotaxis	2,23E-02	4	6,5	6,25	RAC2, IL8, CCR10, IL1B
hsa04620	Toll-like receptor signaling pathway	2,26E-02	3	11,62	4,69	IL8, IL1B, STAT1
GO:0006915	apoptosis	2,49E-02	7	3,03	10,94	IER3, SGK1, TNFSF10, AEN, IL1B, GZMH, STAT1
GO:0012501	programmed cell death	2,66E-02	7	2,98	10,94	IER3, SGK1, TNFSF10, AEN, IL1B, GZMH, STAT1
GO:0019221	cytokine-mediated signaling pathway	2,86E-02	3	11,15	4,69	TXNDC17, IL1B, STAT1
GO:0042981	regulation of apoptosis	3,02E-02	8	2,59	12,5	IER3, TNFSF10, BCLAF1, AEN, NR4A2, IL1B, STAT1, NEFL
GO:0043067	regulation of programmed cell death	3,16E-02	8	2,56	12,5	IER3, TNFSF10, BCLAF1, AEN, NR4A2, IL1B, STAT1, NEFL
GO:0010941	regulation of cell death	3,22E-02	8	2,55	12,5	IER3, TNFSF10, BCLAF1, AEN, NR4A2, IL1B, STAT1, NEFL
GO:0015671	oxygen transport	4,79E-02	2	40,02	3,13	HBA2, HBA1, HBB
GO:0006576	biogenic amine metabolic process	5,17E-02	3	8,05	4,69	SULT1A1, NR4A2, DHPS
GO:0030593	neutrophil chemotaxis	6,58E-02	2	28,91	3,13	IL8, IL1B
GO:0015669	gas transport	6,58E-02	2	28,91	3,13	HBA2, HBA1, HBB
GO:0019835	cytolysis	7,63E-02	2	24,78	3,13	GZMM, GZMH
GO:0045429	positive regulation of nitric oxide biosynthetic process	7,63E-02	2	24,78	3,13	IL1B, HBB
GO:0043065	positive regulation of apoptosis	7,82E-02	5	3,03	7,81	TNFSF10, BCLAF1, AEN, IL1B, STAT1
GO:0043068	positive regulation of programmed cell death	7,98E-02	5	3	7,81	TNFSF10, BCLAF1, AEN, IL1B, STAT1
GO:0010942	positive regulation of cell death	8,08E-02	5	2,99	7,81	TNFSF10, BCLAF1, AEN, IL1B, STAT1
hsa05332:	Graft-versus-host disease	8,83E-02	2	20,06	3,13	HLA-DQB1, IL1B
GO:0008380	RNA splicing	9,14E-02	4	3,66	6,25	PCF11, CLP1, ZRANB2, RNPS1
hsa04940	Type I diabetes mellitus	9,48E-02	2	18,63	3,13	HLA-DQB1, IL1B
GO:0045428	regulation of nitric oxide biosynthetic process	9,70E-02	2	19,27	3,13	IL1B, HBB
GO:0007610	behavior	9,98E-02	5	2,77	7,81	RAC2, IL8, CCR10, NR4A2, IL1B

Supplementary Table 3 : SNP positions and frequencies identified by resequencing in exons and flanking regions of TNFSF10 gene (NM_003810) in the studied population

Position	A1	A2	A2 Frequency (%)	dbSNP number	Location	Frequency class
336	T	C	4,6	rs75278014	Promoter	Rare
380	C	T	15,1	rs12488654	Promoter	Frequent
404	A	G	13,9	rs365238	Promoter	Frequent
465	A	G	0,2	rs149647745	Promoter	Rare
511	A	G	15,1	rs3136586	Promoter	Frequent
536	A	C	1,6	rs146334252	Promoter	Rare
1027	A	G	0,44	rs80208847	Exon (5'UTR)	Rare
1200	C	T	0,44	rs41308132	Exon 1 (non-synonymous)	Rare
1271	A	C	14,6	rs2270418	Intron 1	Frequent
9365	C	T	0,35	rs16845759	Intron 1	Rare
9490	C	A	0,17	rs16845759	Exon 2	Rare
9871	A	T	1,3	rs142625844	Intron 2	Rare
12399	C	A	18,9	rs2241063	Intron 2	Frequent
12609	C	T	0,17	rs146586741	Intron 2	Rare
12656	C	T	0,26	rs55762319	Intron 2	Rare
12969	C	T	2,77	rs3136595	Intron 3	Rare
13726	C	A	18,9	rs3136597	Intron 3	Frequent
15071	A	G	28,13	rs3815496	Intron 3	Frequent
17499	T	A	28,25	rs17848019	Intron 4	Frequent
17534	C	T	0,61	rs114244201	Intron 4	Rare
17655	T	C	0,87	rs56119116	Exon 5 (non-synonymous)	Rare
17868	C	T	0,26	rs145496528	Exon 5 (non-synonymous)	Rare
17967	C	T	28,21	rs1131532	Exon 5	Frequent
18089	C	T	0,17	rs138020036	Exon 5 (3'UTR)	Rare
18192	A	G	1,78	rs41309772	Exon 5 (3'UTR)	Rare
18195	G	A	37,63	rs1131535	Exon 5 (3'UTR)	Frequent
18288	A	G	4,66	rs17600346	Exon 5 (3'UTR)	Rare
18344	C	A	28,22	rs1131542	Exon 5 (3'UTR)	Frequent
18580	G	A	28,09	rs1131568	Exon 5 (3'UTR)	Frequent
18643	G	A	28,22	rs1131579	Exon 5 (3'UTR)	Frequent
18650	C	T	28,13	rs1131580	Exon 5 (3'UTR)	Frequent
18767	C	T	0,51	rs115111869	Exon 5 (3'UTR)	Rare
18783	A	G	0,43	rs150169078	Exon 5 (3'UTR)	Rare
18894	G	A	28,51	rs11720451	3' flanking	Frequent
18900	C	T	0,09	rs138626414	3' flanking	Rare
18921	C	T	0,36	rs149250622	3' flanking	Rare

Supplementary Table 4 : List of qPCR TaqMan assays

Gene	Reporter	AIF Assay ID
ACTB	VIC	Hs99999903_m1
GAPDH	VIC	Hs99999905_m1
RPLP0	VIC	Hs99999902_m1
TNFRSF10a	FAM	Hs00269492_m1
TNFRSF10b	FAM	Hs00366278_m1
TNFRSF10c	FAM	Hs00182570_m1
TNFRSF10d	FAM	Hs00388742_m1
TNFSF10	FAM	HS00234356_m1

Supplementary Table 5 : List of primers used for genotyping of TNFSF10

PCR Forward primer	Sequence	PCR Reverse primer	Sequence
TRAILProPF	ACAACATACAGCTGGGCCAG	TRAILProPR	GTAGTCGTTGGAAAGGAGGG
TRAILE1PF	AAACAGGCCTTGTGCCTATG	TRAILE1PR	GCTTTCATGAAGAGTTGCAATG
TRAILE2PF	TGGCAGAACTGGAAGAGACC	TRAILE2PR	CATCAGCAATGTGGGAAGAA
TRAILE3SF	CCACATTTGGCTGACATCAC	TRAILE3PR	ATGATGGAGGAGGAGGCTTT
TRAILE4PF	GTTTCTCTTTGGACCCTACC	TRAILE4PR	TTTCCTGAGGCCAGTTATGTC
TRAILE5-1PF	CTCCCAACAGTTCCCAATGT	TRAILE5-1PR	CCTTAAGGAAACCTGGAGGC
TRAILE5-2PF	GGGGCCTTTTTAGTTGGCTA	TRAILE5-2PR	CAGCACAACTCAACCCAGAA

Supplementary Table 6 : List of primers used for exons sequencing TNFSF10

SEQ Forward primer	Sequence	SEQ Reverse primer	Sequence
		TRAILE1SR	CCACAGAGAAAGGAAGCAGG
		TRAILE2SR	TTACCCTGTGTGTGCCTCAG
		TRAILE3SR	TGAGCTTCCTAGCTGCCAAT
TRAILE5-1SF	TCCTGGGAATCATCAAGGAG	TRAILE5-1SR	CCTCCTGAAATCGAAAGTATG
TRAILE5-1SF2	GCAGGAACCTCCCAATTTCT		
TRAILE5-2SF	AATCTGAGTAGAGCAGCCAC	TRAILE5-2SR2	TGGCATGATCTCACCACACT

Supplementary Figure 1

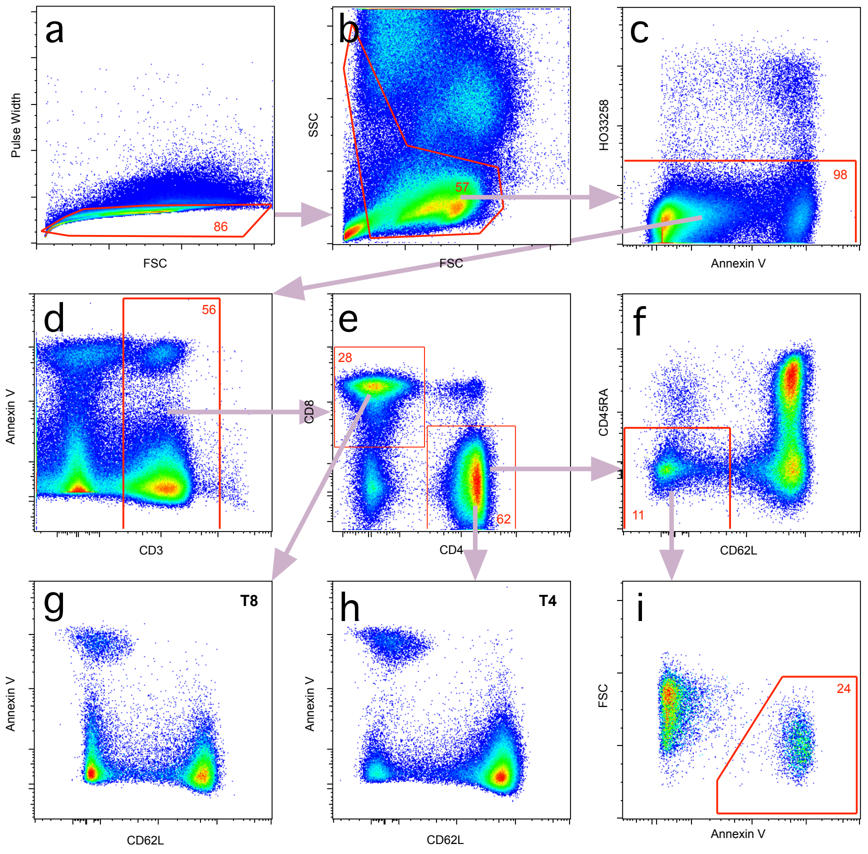

Gating strategy for the quantification of AnnexinV-positive cells in T4EM, 18 hours after irradiation at 2Gy of a PBMC sample.

Data was analyzed using FlowJo (v9.6.4).

Exclusion of doublets using a gate on FSC vs Pulse Width (panel a).
Lymphocyte gating using a large scattergate, shaped to include apoptotic lymphocytes (lower FSC, higher SSC; panel b).
Exclusion of dead cells on the basis of HO33258 fluorescence (panel c).
T lymphocytes were identified on the basis of positive PETxR (CD3) fluorescence (panel d).
Identification of T4- and T8 lymphocytes through PECy7 (CD4) vs APCH7 (CD8) fluorescence (panel e).
APC (CD45RA) vs PE (CD62L) histograms to identify naïve (N; CD62L+CD45RA+), central memory (CM; CD62L+CD45RA-), effector memory (EM; CD62L-CD45RA-), and terminal effector (TE; CD62L-CD45RA+) T4 and T8 (panel f).
The proportion of apoptotic cells was finally determined by application of an identical gate on a bivariate FSC vs FITC (AnV) to all of the identified subpopulations (exemplified for T4EM; panel i).

Dot plots show absence of AnnexinV positive cells in T8- and T4 CD62L expressing lymphocytes (panels g and h, respectively).

Supplementary Figure 2

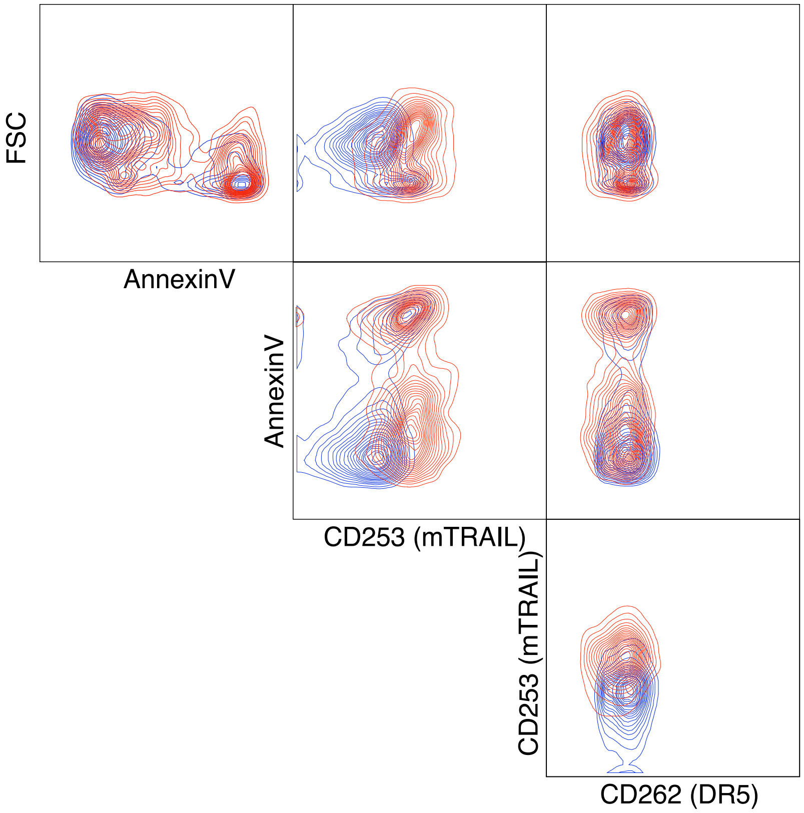

Higher expression of surface bound TRAIL (mTRAIL; CD253) in a sensitive sample (red contours) as compared to a resistant sample (blue contours).

T4EM lymphocytes were gated according to the strategy outlined in Supplementary Figure 1. Overlayed NbyN representation of contour plots of T4EM lymphocytes for FSC, AnnexinV, CD253 (TRAIL), and CD262 (DR5), illustrating increased expression of mTRAIL in sensitive samples.
